# MicroRNAs as T Lymphocyte Regulators in Multiple Sclerosis

**DOI:** 10.3389/fnmol.2022.865529

**Published:** 2022-04-25

**Authors:** Lin Wang, Yuanyuan Liang

**Affiliations:** Department of Emergency Medicine, Shengjing Hospital of China Medical University, Shenyang, China

**Keywords:** MicroRNAs, T lymphocytes, multiple sclerosis, pathogenesis, biomarkers, therapy

## Abstract

MicroRNA (miRNA) is a class of endogenous non-coding small RNA with regulatory activities, which generally regulates the expression of target genes at the post-transcriptional level. Multiple Sclerosis (MS) is thought to be an autoimmune-mediated chronic inflammatory demyelinating disease of the central nervous system (CNS) that typically affect young adults. T lymphocytes play an important role in the pathogenesis of MS, and studies have suggested that miRNAs are involved in regulating the proliferation, differentiation, and functional maintenance of T lymphocytes in MS. Dysregulated expression of miRNAs may lead to the differentiation balance and dysfunction of T lymphocytes, and they are thus involved in the occurrence and development of MS. In addition, some specific miRNAs, such as miR-155 and miR-326, may have potential diagnostic values for MS or be useful for discriminating subtypes of MS. Moreover, miRNAs may be a promising therapeutic strategy for MS by regulating T lymphocyte function. By summarizing the recent literature, we reviewed the involvement of T lymphocytes in the pathogenesis of MS, the role of miRNAs in the pathogenesis and disease progression of MS by regulating T lymphocytes, the possibility of differentially expressed miRNAs to function as biomarkers for MS diagnosis, and the therapeutic potential of miRNAs in MS by regulating T lymphocytes.

## Introduction

Multiple sclerosis (MS), an autoimmune neurological disease of the central nervous system (CNS), which is predominantly characterized by diffuse demyelinating lesions of the white matter (Yamout and Alroughani, 2018), but can also affect the gray matter (Klaver et al., 2013). T lymphocytes play a central role in cell-mediated immunity and can be divided into two main subgroups: (1) CD4 + T helper lymphocytes that regulate the quality and degree of immune response by releasing cytokines and that secrete proteins that can affect cellular functions related to antibacterial responses. In contrast, (2) CD8 + T cytotoxic lymphocytes have the ability to directly recognize and kill infected or transformed cells. The loss of balance between T cell subsets, resulting in attacks on self-antigenic myelin basic protein (MBP), is the most direct known cause of MS, but more details and mechanisms remain to be revealed (Deng et al., 2019; Lückel et al., 2019; Schorer et al., 2019). A large number of studies have demonstrated that CD4 + T cells play an important role in the pathogenesis of MS; helper T cell 1 (Th1) and Th17 cells are involved in the pathogenesis of MS, and regulatory T cells (Tregs) are involved in the pathogenesis of MS in experimental autoimmune encephalomyelitis (EAE) models (Chitnis, 2007; Basak and Majsterek, 2021). Tregs and interleukin-10 (IL-10) are two important negative regulators of disease progression (Ma et al., 2009). In addition, an increase in the number of Th2 cells or the inhibition of Th1/Th17 cells may delay the progression of EAE (Haghmorad et al., 2021; Hou et al., 2021). Thus, at the molecular level, T cell proliferation and function are regulated by a complex network of transcriptional and post-transcriptional mechanisms, including transcription factors, signaling molecules, epigenetic modifications, and microRNA (miRNA) alterations (Okoye et al., 2014; Kroesen et al., 2015).

MicroRNAs are small non-coding RNAs with the capability of regulating gene expression at the post-transcriptional level either by inhibiting messenger RNA (mRNA) translation or by promoting mRNA degradation (Correia De Sousa et al., 2019). In general, a specific miRNA can regulate several mRNA transcripts, and different transcripts can participate in different cellular programs, while one mRNA transcript may be regulated by multiple miRNAs (Matkovich et al., 2013). In the CNS, miRNAs are abundant and can affect development, proliferation, differentiation, plasticity, and other cellular processes (Iyengar et al., 2014). MiRNAs are also highly expressed in immune cells and are involved in both innate and adaptive immune responses (Zhong et al., 2018). It has been reported that miRNA is a key regulatory factor to maintain immune tolerance (Scalavino et al., 2020). In the process of miRNA synthesis, the deletion of Dicer and Drosha enzymes can lead to T cell dysfunction and autoimmune diseases (Landskroner-Eiger et al., 2013). In different immune cell subgroups, miRNA transcription is different, indicating that the functions of naïve, effector, and memory T cells and regulatory T cells depend on miRNA regulation (Podshivalova and Salomon, 2013; Zhang et al., 2016). Recent studies have found that there is a specific expression pattern of miRNAs in the pathogenesis of MS, and the abnormal expression of miRNAs may be the “priming factor” that leads to the pathogenesis of MS; this pathogenic effect is likely to be achieved by regulating the activation of T cells (De Santis et al., 2010; Wei and Pei, 2010). In addition, miRNAs are histologically specific, can be expressed in paracrine forms, and are detected in many different biological fluids [cerebrospinal fluid (CSF), serum, urine, and saliva] (Jin et al., 2013). Therefore, peripheral circulating miRNAs may be used as biomarkers for the diagnosis of MS, the discrimination of MS subtypes, and the prediction of MS prognosis. Moreover, in-depth studies of miRNA regulation of T lymphocytes involved in the pathogenesis of MS may help to develop new strategies for the treatment of MS at the transcriptional and post-transcriptional levels.

Here, by summarizing recent reports, we discuss the role of T lymphocyte dysfunction in the pathogenesis of MS. Dysregulated expression of miRNAs in T lymphocytes directly or indirectly affects T lymphocyte function and, thus, is involved in the pathogenesis of MS. These miRNAs may be differentially expressed in peripheral circulation; in light of the current data, we further explore the potential of these miRNAs in MS diagnosis and distinguishing MS subtypes and targeting differentially expressed miRNAs to provide novel therapeutic strategies for MS patients.

## T Lymphocyte Subsets and Pathogenesis of Multiple Sclerosis

T lymphocytes are derived from lymphoid stem cells from the bone marrow. After differentiation and maturation in the thymus, they are distributed to immune organs and tissues of the whole body through lymph and blood circulation to play an immune function (Themeli et al., 2013). Generally, T lymphocytes can be divided into CD4+ and CD8+ subgroups according to the differentiation of antigens on the cell surface. CD4 + T lymphocytes recognize exogenous antigen peptides presented by major histocompatibility complex (MHC)-II molecules and differentiate into Th cells after activation. CD8 + T lymphocytes recognize endogenous antigen peptides presented by MHC-I molecules; after activation, they mainly differentiate into cytotoxic T lymphocytes (Parkhurst et al., 2004; Afridi et al., 2016). Primary CD4 + T lymphocytes have been induced to differentiate into Th1 cells by interferon γ (IFN-γ), and differentiate into Th17 cells by transforming growth factor-β (TGF-β) and interleukin-6 (IL-6) (Van Hamburg et al., 2008).

CD4 + T lymphocytes play a key role in initiating the autoimmune response in MS patients (Kaskow and Baecher-Allan, 2018). It is generally believed that cytokines secreted by Th1 cells, such as IFN-γ and tumor necrosis factor-β (TNF-β), can activate macrophages to destroy oligodendrocytes, resulting in pathological myelination; IFN-γ in turn induces the production of Th1 cells (Merrill, 1992; Næss et al., 2001). TNF-α and IL-12 have proinflammatory effects, and TNF-α can directly produce cytotoxic effects on oligodendrocytes (Dopp et al., 1997). IL-12 is involved in the regulation of T lymphocyte responses and may be associated with the pathogenesis of MS (Gran et al., 2004). In addition, some measures to delay the MS process by inhibiting Th1 cells have also been shown to be effective (Xu et al., 2021). CD4+ Th2 cells are lymphocytes of the anti-inflammatory family and include major secretion immune adjustment factors such as IL-4, IL-5, IL-6, and IL-10 (Bui et al., 2017).

CD8 + T lymphocytes were found to be present in MS plaques, and these cells accumulated over time and were more numerous than CD4 + T lymphocytes (Lassmann, 2018). Human leukocyte antigen (HLA)-E-restricted CD8+ regulatory T lymphocytes can be induced by IFN-γ, which can kill immune cells such as CD4 + T lymphocytes and induce other cells to secrete some inhibitory factors such as TGF-β; IL-10 suppresses immune function and maintains disease stability in MS patients in remission (Frisullo et al., 2010). CD8 + regulatory T cell cloning in the blood and CSF of relapsing-remission MS (RRMS) patients is significantly less than that of convalescent MS patients. In addition, CD94/NKG2A killer suppressor receptors inhibit CD8+ regulatory T cell cloning and killing of other immune cells, exacerbating the disease in RRMS patients (Correale and Villa, 2008; Uemura et al., 2008). All these suggest the role of CD8+ regulatory T lymphocytes in the pathogenesis of MS. Correale et al. suggested the induction of CD8 + CD25 + Foxp3 + cells, CD4+ self-reactive cells, IFN-γ, and IL-17 in the culture plate was inhibited, and the clone of CD8+ regulatory T lymphocytes in the blood and CSF of MS patients in the exacerbation stage is significantly lower than that of patients in the remission stage, indicating that CD8+ regulatory T lymphocytes play a significant regulatory role in MS and may stop the progression of MS (Correale and Villa, 2010). Among MS patients treated with glucocorticoid, Aristimuño et al. (2008) found that the number of CD4+ and CD8+ regulatory T lymphocytes increased, while the number of CD8+ effector and memory T lymphocytes had a downward trend, indicating that CD8+ regulatory T lymphocytes may play a role in preventing the progression of MS, while CD8+ effector T lymphocytes promote the progression of MS. Effector T lymphocytes may attack the myelin sheath of oligodendrocytes by releasing granzymes, TNF, cytolysin, and other inflammatory mediators and may cause apoptosis of myelin cells (Aristimuño et al., 2008).

Th17 lymphocytes are a newly discovered subgroup of Th lymphocytes, named for their specific secretion of IL-17. Their main function is to prevent the infection of extracellular bacteria by regulating immune cells or non-immune cells, and they also play an important role in the pathogeneses of autoimmune diseases (Miossec and Kolls, 2012). The number of Th17 lymphocytes in the CSF of RRMS patients was found to increase significantly during the relapse stage, suggesting that Th17 lymphocytes may play a pathogenic role in MS (Brucklacher-Waldert et al., 2009). Th17 lymphocytes release proinflammatory mediators including IL-17A, which can downregulate the expression of blood-brain barrier (BBB) connexin, increase the penetration of BBB, and facilitate the entry of soluble inflammatory molecules and other circulating immune molecules into the CNS (Rahman et al., 2018). The level of IL-17 in the BBB of MS patients is associated with the destruction of BBB, suggesting that IL-17 has similar pathogenicity for EAE and MS (Setiadi et al., 2019). In addition, IL-17 inhibits remyelination and repair and promotes oligodendrocyte apoptosis (Pareek et al., 2011).

Treg is another important subgroup derived from the differentiation of CD4 + T lymphocytes under the stimulation of TGF-β. There are at least two types of CD4+ Tregs: CD4 + CD25 + Tregs (nTregs) naturally produced by the thymus, and Tregs produced by peripheral induction (iTregs). NTregs mainly inhibit inflammation in a cell-contact dependent manner, while iTregs play an immunosuppressive role by secreting the inhibitory cytokines IL-10 or TGF-β (Bluestone and Abbas, 2003; Moaaz et al., 2019). The possible mechanisms of Tregs involved in the pathogenesis of MS include the decrease of cell number, the loss of inhibitory ability, and a defect in the ability to migrate to the CNS (Abdolahi et al., 2015). The decrease of FoxP3 expression in Tregs of MS patients suggests that the inhibitory function of Tregs is reduced (Etesam et al., 2016). Studies have shown that increasing the number of Tregs in EAE mice can inhibit the migration and infiltration of mouse autoreactive T lymphocytes into the CNS and significantly improve the symptoms of EAE induced by myelin oligodendrocyte glycoprotein (Yan et al., 2010).

NKT cells are classically described as a subset of T cells sharing characteristics of NK cells and typical αβ T cells (Zarobkiewicz et al., 2021). NKT cells recognize lipid and glycolipid antigens presented in the context of CD1d molecules, a non-classical MHC molecule (Shinjo et al., 1987). Typically, NKT cells are divided into three distinct populations-classical type I NKT (termed also invariant NKT, iNKT), type II (non-classical) NKT, and NKT-like cells (Godfrey et al., 2004). There was a great reduction of iNKT cells in the peripheral blood of MS patients (Illés et al., 2000). After treatment with IFN-β, the number and function of iNKT cells recovered (Gigli et al., 2007). In the remission stage of MS patients, iNKT cells produced more IL-4 and showed Th2-type response polarization, suggesting that iNKT cells may play an immunomodulatory role through Th2 type response in MS patients (Araki et al., 2003). In EAE mice, iNKT cells mainly secreted IL-4 and IL-10 to inhibit EAE (Singh et al., 2001). In addition, iNKT cells can also inhibit the further deterioration of EAE by regulating the polarization of macrophages and the proliferation of myeloid-derived suppressor cells (Condamine and Gabrilovich, 2011).

Innate immune CD16 + γδT lymphocytes also play a role in the pathogenesis of MS. *In vitro* experiments have shown that CD16 + γδT lymphocytes can induce cytotoxicity directly to the CNS. In addition, CD16 + γδT lymphocytes can produce antibody-dependent cytotoxicity to the antibody-coated target cells, thereby damaging the myelin sheath; however, the specific mechanisms have not been fully elucidated (Chen and Freedman, 2008a,b).

Various subtypes of T lymphocytes are involved in the pathogenesis and development of MS. They play damaging, regulatory, or protective roles and even have several simultaneous roles in different disease stages. Further studies of the immune role of T lymphocyte subsets in the pathogenesis of MS may provide new ideas for the treatment of MS. In recent years, as miRNA regulation of the immune system and involvement in the pathogenesis of autoimmune diseases have been reported, people have realized that miRNAs not only have a specific expression pattern in the course of MS, but may also be an important factor leading to the pathogenesis of MS (Wu and Chen, 2016). There are specific distributions of miRNAs transcriptomes in different subtypes of T lymphocytes, and they play a very important regulatory role in the development and differentiation of CD4 + T lymphocytes, the activation of effector T lymphocytes, the maintenance of the immune tolerance function of Treg cells, and the production of memory T lymphocytes. Inhibiting the function of miRNAs or changing the expression of miRNAs can lead to abnormal functions of T lymphocytes, which in turn participates in the pathogenesis and progression of MS.

## MicroRNAs Are Involved in Multiple Sclerosis by Regulating T Lymphocytes

MicroRNAs are a newly discovered class of non-coding single-stranded RNA molecules that act mainly at the post-transcriptional level. They bind to target genes and induce complete or partial degradation of mRNAs and thus regulate gene expression (Lindberg et al., 2010). miRNAs play an important regulatory role in maintaining immune homeostasis and normal immune function, and their dysregulated expression may be one of the important reasons for disrupting the immune tolerance balance. The abnormal expression of miRNAs in T lymphocytes of MS patients is key to initiating the autoimmune pathogenesis process of MS, which mainly involves the Th1, Th17, Treg, and CD8+ lymphocytes (Bennett and Stüve, 2009).

Th1 cells mediate autoimmune demyelination in MS. The expression of miR-27b and miR-128 is increased in naïve CD4 + T lymphocytes, and miR-340 expression is increased in memory CD4 + T lymphocytes in MS patients; these increased miRNAs contribute to the proinflammatory Th1 response by inhibiting the expression of IL-4 and B lymphoma Mo-MLV insertion region 1 homolog (BMI1). Downregulation of expression of these miRNAs with oligonucleotide miRNA inhibitors leads to the restoration of Th2 responses and has therapeutic potential in regulating T-cell phenotypes in MS (Guerau-De-Arellano et al., 2011). miR-142-5p expression is increased in the frontal white matter of MS patients; overexpression of miR-142a-5p in activated lymphocytes could shift the pattern of T lymphocyte differentiation toward Th1 lymphocytes. Thus, miR-142-5p may be involved in the pathogenesis of autoimmune neuroinflammation by promoting Th1 lymphocyte differentiation (Talebi et al., 2017). The expression of miR-155 is increased in the serum of MS patients, especially during the relapse period; miR-155 enhances the differentiation of Th17 and Th1 cells by increasing the cytokine production of both IL-17A and IFN-γ, which sustain the inflammation response and aggravate clinical signs of the EAE model (Zhang et al., 2014). miR-140-5p may be involved in the pathogenesis of MS by regulating encephalitogenic T cells. Another mechanism study found that miR-140-5p expression is significantly decreased in MS patients; signal transducer and activator of transcription 1 (STAT1) is a functional target of miR-140-5p, and overexpression of miR-140-5p suppresses phenomenological Th1 differentiation by inhibiting the activation of STAT1 and the expression of its downstream target, T-bet (Guan et al., 2016). miR-92a is one of the substantially upregulated miRNAs in MS. Rezaei et al. (2019) demonstrated that miR-92a expression is significantly enhanced at the peak of EAE, accompanied by decreased expression of DUSP10 or TSC1. Another study found that miR-92a might promote Th1 differentiation, likely owing to downregulation of DUSP10 and TSC1 expression (Rezaei et al., 2019). miR-29b expression is increased in T cells from MS patients and EAE mice. Using mice deficient in miR-29, Wan et al. (2019) suggested that miR-29ab1 is critical in regulating Th1 differentiation through repression of T-bet and IFN-γ (Smith et al., 2012). miR-182 is upregulated in RRMS patients and is correlated with increased numbers of CD4 + Th1 cells and IFN-γ production in the circulation. In EAE mice, overexpression of miR-182 resulted in exacerbation of clinical symptoms and augmentation of Th1 and Th17 differentiation (Wan et al., 2019).

Invasion of Th17 cells into the CNS is an underlying pathogenic mechanism in MS (Abarca-Zabalía et al., 2020). A disturbed Th17/Treg balance contributes to the development of autoimmune diseases, including EAE and MS (Zhou et al., 2016). It was reported that the expression of miR-27a is upregulated, while miR-214 expression is downregulated in relapsing-phase MS patients; the two miRNAs play inhibitory and promoting roles in Th17 differentiation by modulating TGF-β and mTOR signaling, respectively (Ahmadian-Elmi et al., 2016). Li et al. observed that miR-1-3p expression is upregulated in Th17 cells from MS-relapse patients. Overexpression of miR-1-3p in naïve CD4 + T cells promotes Th17 cell differentiation by increasing ETS1 expression (Li et al., 2020). Azimi et al. (2019) demonstrated that miR-326 is involved in the immunopathogenesis of MS by inducing Th17 cell differentiation and maturation. Azimi et al. suggested miR-326 negatively regulates differentiation of naïve T to Th17 cells by targeting Ets-1 (Junker et al., 2009; Azimi et al., 2019).

miR-326 may also target the CD46 molecule, which could increase the degradation of myelin by inhibiting the phagocytic activity of macrophages, thereby increasing the severity of MS and EAE (Junker et al., 2009). miR-155 expression is significantly upregulated in brain-infiltrating myelin-autoreactive CD4 + T lymphocytes, can promote Th17 (but not Th1) development by targeting two heat shock protein genes, *Dnaja2* and *Dnajb1*, and contributes to the development of EAE (Mycko et al., 2015). In *in vitro* cytokine-induced Th17 cells, miR-21 and miR-181c expression is significantly increased, and their upregulation promotes Th17 cell differentiation by targeting Smad7. In miR-21 or miR-181c-deficient mice, Th17 cell differentiation is defective, and EAE progresses slowly. However, because the two miRNAs act on the same target gene, whether their mechanism of promoting Th17 cell differentiation is competitive or synergistic needs to be further verified (Murugaiyan et al., 2015; Zhang et al., 2018; Huang et al., 2021). miR-26a is another important regulator for balancing Th17/Treg differentiation. It was reported the miR-26a expression is downregulated in MS patients and C57BL/6 mice in an EAE model system; decreased miR-26a resulted in increased expression of Th17-related cytokines and vice versa. By contrast, expression of Foxp3, the Treg cell-specific transcription factor, was found to be positively correlated with miR-26a expression. Further study found that overexpression of miR-26a could inhibit Th17 and promote Treg cell function by targeting IL-6 (Zhang et al., 2015). Tob1 is a well-known suppressor of Th17 differentiation and is a direct target of miR-590. Liu Q. et al. (2017) revealed that miR-590 expression is markedly increased in Th17 cells of MS patients and can promote pathogenic Th17 differentiation through inhibiting Tob1 expression. The expression of let-7f-5p is significantly downregulated in CD4 + T lymphocytes from MS patients and during the process of Th17 differentiation. Li et al. suggested that overexpression of let-7f-5p could inhibit Th17 differentiation. Signal transducer and activator of transcription 3 (STAT3) is a direct target of let-7f-5p and also a critical transcription factor of Th17 cells; let-7f-5p may serve as a potential inhibitor of Th17 differentiation in the pathogenesis of MS by targeting STAT3 (Li et al., 2019). Consistent results were obtained by Angelou et al. (2019), who re-emphasized the pivotal roles of let-7 in clonal expansion, and acquisition of the pathogenic Th17 phenotype and suggested that let-7 may directly target the chemokine receptors CCR2 and CCR5 as well as the cytokine receptors IL-1R1 and IL-23R to inhibit pathogenic Th17 differentiation during EAE development.

miR-17 and miR-19b are two miRNAs responsible for promoting Th17 responses. Liu et al. (2014) found miR-17 enhances Th17 polarization by inhibiting Ikaros family zinc finger 4 (IKZF4), whereas miR-19b reduces the expression of phosphatase and tensin homology (PTEN), thereby activating the PI3K-AKT-mTOR axis crucial for Th17 differentiation. miR-146a deficiency can induce more severe EAE in mice and cause increased differentiation into Th17 cells; a mechanical study suggested that miR-146a is an important molecular brake that blocks the autocrine IL-6- and IL-21-induced Th17 differentiation pathways in autoreactive CD4 T cells (Li et al., 2017). Upregulated let-7e expression was correlated with the development of EAE; inhibiting let-7e *in vivo* reduced the number of encephalitogenic Th1 and Th17 cells and attenuated EAE (Guan et al., 2013). miR-141 and miR-200a are negative regulators of Th17 cell differentiation in RRMS patients; in the relapsing phase of MS, the expression of both miR-141 and miR-200a shows upregulation, which is involved in the pathogenesis of MS by inducing differentiation of Th17 cells and inhibiting differentiation to Treg cells (Naghavian et al., 2015). Wu et al. (2017) showed that miR-448 can enhance Th17 differentiation and aggravate the disease; protein tyrosine phosphatase non-receptor type 2 (PTPN2) is an anti-inflammatory regulator with the capacity to suppress Th17 differentiation and is a direct target of miR-448, which in turn might promote Th17 differentiation in MS by inhibiting PTPN2.

miR-30a expression is greatly decreased during Th17 differentiation in MS patients and EAE mice; IL-21R is a direct target of miR-30a, and overexpression of miR-30a results in fewer Th17 cells and alleviated EAE by targeting IL-21R (Qu et al., 2016). miR-15b is another important miRNA that regulates the differentiation of Th17 cells. miR-15b expression is significantly downregulated in MS patients and EAE mice. By regulating o-GlCNAC transferase activity, miR-15b suppresses Th17 differentiation both *in vivo* and *in vitro* and is involved in the pathogenesis of MS (Liu R. et al., 2017). miR-183c expression is increased in Th17 cells and is induced by the IL-6-STAT3 signal; it can promote pathogenic cytokine production during Th17 cell development and enhances autoimmunity. In fact, miR-183c directly inhibits the expression of the transcription factor Foxo1, which negatively regulates the pathogenicity of Th17 cells by inhibiting the expression of IL-1R1 (Ichiyama et al., 2016).

Tregs dysregulation is a common phenomenon in autoimmune diseases including MS. TGF-β signaling is essential for the development and function of Tregs. The levels of TGF-β signaling components in naïve CD4 T cells of MS patients are reduced (Aram et al., 2020). In an miRNA profile study of naïve CD4 + T lymphocytes in MS patients, Severin et al. (2016) identified 12 differentially expressed miRNAs that were validated using qRT-PCR and predicted to target the TGF-β signaling pathway, including: miR-18a, -27b, -103a, -128, -141, -212, -500a, -628-3p, -708, let-7a, -7b, and -7f. They inferred that miRNAs may be one important reason for Tregs defects observed in MS patients. The increased expression of a variety of TGF-β-targeting miRNAs in naïve CD4 + T cells of MS patients impairs TGF-β signaling and inhibits the development of Tregs, thereby increasing the susceptibility to MS. Another miRNA genome-wide expression profile by microarray analysis on CD4 + T lymphocytes showed the miR-25 and miR-106b expression is downregulated in RRMS patients; these two miRNAs may regulate the TGF-β signaling pathway and Tregs differentiation and maturation by modulating CDKN1A/p21 and BCL2L11/Bim (De Santis et al., 2010). miR-30d expression is increased in feces from EAE mice and untreated MS patients. Liu et al. (2019) showed that synthetic miR-30d can ameliorate EAE through expansion of Tregs. Let-7i expression is upregulated in MS patients; upregulated let-7i expression inhibits CD4 + T lymphocyte differentiation into Treg cells by decreasing the expression of transforming growth factor β receptor 1 (TGFBR1) and insulin-like growth factor 1 receptor (IGF1R) (Kimura et al., 2018). It has been reported the miR-27 expression is highly upregulated in T cells isolated from MS patients. Cruz et al. (2017) showed that miR-27 negatively regulates Treg cells; mechanically, the excessive expression of miR-27 could negatively impact FOXP3 induction and Treg development through targeting of c-Rel (Ruan et al., 2009). Smad7 is also a direct target of miR-181a and -b, and its expression is significantly decreased in brain white matter from MS patients as well as in the spinal cords of EAE mice during the acute and chronic phases of the diseases. Overexpression of miR-181a and -b-inhibited Th1 generation in CD4 + T cells promotes Treg differentiation, providing potential therapeutic options for controlling inflammation in MS (Ghorbani et al., 2017).

**FIGURE 1:**
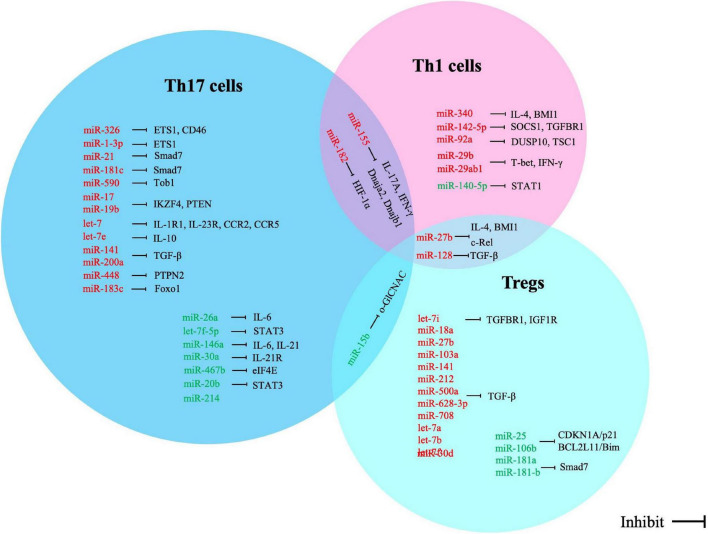
Correlation between miRNA alterations and expression of direct targets in T cell subsets of multiple sclerosis. Red words indicate the up-regulated miRNAs and green words indicate the down-regulated miRNAs.

Based on our review of recent literature, we believe that miRNAs are involved in the process of T lymphocyte differentiation, activation, and function through a variety of pathways. These mRNAs (Figure 1 and Table 1) are involved in the regulation of T lymphocyte differentiation and transformation, such as the balance between Th1/Th2 and Th17/Tregs. T lymphocytes phenotypic disorders are the main pathological mechanism of a variety of autoimmune diseases, including MS. Importantly, in view of T-cell-specific miRNA expression patterns, evaluation of differential miRNA expression may enable these miRNAs to become diagnostic biomarkers for MS as an important complement to current diagnostic methods (Paul et al., 2019).

**TABLE 1:** Dysregulation of miRNAs in T sub-typing cells of multiple sclerosis.

Subtypes of T cells	miRNAs	Models/patients	Target	Function	References
Th1 cells	miR-27b, miR-128, miR-340	MS mice/patients	IL-4, BMI1	Contribute to pro-inflammatory Th1 response.	Guerau-De-Arellano et al., 2011
	miR-142-5p	MS patients	SOCS1, TGFBR1	Promote Th1 lymphocytes differentiation	Talebi et al., 2017
	miR-155	MS patients/EAE	IL-17A, IFN-γ	Promote Th1 and Th17 lymphocytes differentiation	Zhang et al., 2014
	miR-140-5p	MS patients	STAT1	Suppress phenomenological Th1 differentiation	Guan et al., 2016
	miR-92a	EAE mice	DUSP10, TSC1	Promote Th1 lymphocytes differentiation	Rezaei et al., 2019
	miR-29b	MS patients/EAE mice	T-bet, IFN-γ	Regulate the Th1 differentiation	Smith et al., 2012
	miR-182	RRMS patients	HIF-1α	Promote Th1 differentiation	Wan et al., 2019
Th17 cells	miR-326	RRMS patients	ETS1, CD46	Regulate differentiation of naïve T to Th17 cells	Junker et al., 2009
	miR-1-3p	MS-relapse patients	ETS1	Promote Th17 cells differentiation	Li et al., 2020
	miR-155	EAE mice	Dnaja2, Dnajb1	Promote Th17 cells development	Mycko et al., 2015
	miR-21	EAE mice/cell models	Smad7	Promote Th17 cells differentiation	Murugaiyan et al., 2015
	miR-181c	EAE mice/cell models	Smad7	Promote Th17 cells differentiation	Zhang et al., 2018
	miR-26a	MS patients/C57BL/6 mice	IL-6	Downregulate Th17 and to upregulate Treg cell function	Zhang et al., 2015
	miR-590	MS patients	Tob1	Promote pathogenic Th17 differentiation in MS and enhance inflammation in CNS	Liu Q. et al., 2017
	let-7f-5p	MS patients	STAT3	Inhibit Th17 differentiation	Li et al., 2019
	miR-17, miR-19b	EAE mice	IKZF4, PTEN	Enhance Th17 polarization and differentiation	Liu et al., 2014
	let-7	EAE mice	Il1r1, Il23r, Ccr2, Ccr5	Inhibit the pathogenic Th17 differentiation	Angelou et al., 2019
	miR-146a	EAE mice	IL-6, IL-21	Regulate Th17 differentiation	Li et al., 2017
	let-7e	EAE mice	IL-10	Promote pathogenic Th17 differentiation	Guan et al., 2013
	miR-141, miR-200a	RRMS patients	TGF-β	Induce differentiation of Th17 cells	Naghavian et al., 2015
	miR-448	MS patients	PTPN2	Promote pathogenic Th17 differentiation	Wu et al., 2017
	miR-30a	MS patients/EAE mice	IL-21R	Inhibit differentiation of Th17 cells	Qu et al., 2016
	miR-467b	EAE mice	eIF4E	Suppress Th17 cell differentiation	Wu et al., 2021
	miR-15b	MS patients/EAE mice	o-GlCNAC	Suppress Th17 cell differentiation	Liu R. et al., 2017
	miR-20b	MS patients/EAE mice	STAT3	Inhibit differentiation of Th17 cells	Zhu et al., 2014
	miR-183c	EAE mice	Foxo1	Promote the pathogenic cytokines production during Th17 cell development	Ichiyama et al., 2016
Tregs	miR-18a, -27b, -103a, -128, -141, -212, -500a, -628-3p, -708, let-7a, -7b, -7f	MS patients	TGF-β	Impair TGF-β signaling and inhibits the development of Tregs.	Severin et al., 2016
	miR-30d	EAE mice	–	Expansion of Tregs	Liu et al., 2019
	let-7i	MS patients	TGFBR1, IGF1R	Inhibited CD4 + T lymphocytes differentiation into Tregs	Kimura et al., 2018
	miR-25, miR-106b	RRMS patients	CDKN1A/p21, BCL2L11/Bim	Disrupt the TGF-β signaling pathway and inhibit Tregs differentiation and maturation	De Santis et al., 2010
	miR-27	MS patients	c-Rel	Inhibit Treg development	Cruz et al., 2017
	miR-181a, 181-b	MS patients/EAE	Smad7	Influence differentiation of Tregs	Ghorbani et al., 2017

## MicroRNAs in T Lymphocytes as Multiple Sclerosis Diagnostic Biomarkers

The dysregulated expression of miRNAs is involved in the pathogenesis of MS through modulating the T lymphocyte phenotype. According to MS complex pathophysiology and innate immunity as well as adaptive immunity contribution to disease, a changed expression profile of miRNAs of a specific T-cell subgroup may provide new potential biomarkers for MS (Table 2).

**TABLE 2:** Studies of diagnostic or prognostic biomarkers in patients of multiple sclerosis.

MIRNAs	Expression changes	T cell subtypes	Samples	Biomarker type and indication	References
miR-21-5p, -26b-5p, -29b-3p, -142-3p, -155-5p	Down-regulated	CD4 + T cells	12P/12HC	Diagnosis biomarker for SPMS	Sanders et al., 2016
miR-1-3p	Up-regulated	Th17 cells	36P/33HC	Diagnosis and severity biomarker for MS	Li et al., 2020
miR-590	Up-regulated	Th17 cells	42P/33HC	Diagnosis biomarker for MS	Liu R. et al., 2017
miR-30, -34a	Up-regulated	CD4 + T cells	40P/20HC	Diagnosis biomarker and discriminate for relapsing and remitting phases in RRMS patients	Ghadiri et al., 2018
miR-199a	Down-regulated				
miR-21	Down-regulated	CD4 + T cells	20P/12HC	Discriminate for relapsing and remitting phases in RRMS patients, and SPMS patients	Ruhrmann et al., 2018
miR-26a, miR-326	Up-regulated	Th17 cells	40P/20HC	Discriminate for relapsing and remitting phases in RRMS patients	Honardoost et al., 2014
miR-155	Down-regulated	CD8 + T cells	25P/10HC	Diagnosis biomarker for MS	Elkhodiry et al., 2021
miR-1, -20a, -28, -95, -146a, -335, -625	Down-regulated	PBMC	12 third trimester/12 post-partum/12P	Monitor disease activity of pregnancy in MS patients	Søndergaard et al., 2020

*MS, multiple sclerosis; P, Patients; PBMC, peripheral blood mononuclear cells; HC, healthy controls; SPMS, secondary progressive MS, RRMS, remitting relapsing MS.*

A case-control study was performed by Ghadiri et al., and forty RRSM patients were enrolled (including 20 relapsing and 20 remitting MS patients); they detected the expression levels of several miRNAs in CD4 + T cells using RT-PCR and further evaluated the diagnostic value of these miRNAs for MS. The results showed that the expression levels of miR-30c and miR-34a are elevated significantly in relapsing MS patients compared with those of remitting ones and healthy controls, whereas the miR-199a expression levels were higher in the remitting patients than the relapsing ones and healthy controls. In addition, the transcript level of miR-19a was increased in relapsing patients versus remitting patients, but there was no meaningful difference between MS patients and healthy controls. Receiver operating characteristics (ROC) analysis suggested the expression levels of miR-19a, -30c, and -34a have a discriminable value for relapsing and remitting phases in RRMS patients (Ghadiri et al., 2018).

The increase in miR-21 level usually represents an inflammatory response (Loboda et al., 2016). In MS, miR-21 expression was found to be upregulated in RRMS patients in relapse. However, in remitting phase, the expression levels of miR-21 were decreased in CD4 + T cells of RRMS patients, which could be used as a diagnostic biomarker to help distinguish relapsing and remitting phases in RRMS patients. Moreover, Ruhrmann et al. (2018) observed lower expression of miR-21 in RRMS patients than in secondary progressive MS (SPMS) patients and controls, and this difference became significant in a larger independent cohort. miR-155 expression was downregulated in CD8 + T cells of RRMS patients and was associated with the patients’ Expanded Disability Status Scale (EDSS), shedding light on the potential use of miR-155 in the diagnosis of MS (Elkhodiry et al., 2021). miR-26a and miR-326 expression was also upregulated in peripheral blood lymphocytes of relapsing phase MS patients when compared with that of the remitting-phase patients and healthy controls. Furthermore, miR-326 was confirmed as a biomarker to discriminate between relapsing and remitting phases of MS with high specificity and sensitivity (100%), as determined by ROC analysis (Honardoost et al., 2014).

Sanders et al. (2016) utilized next-generation sequencing (NGS) for the miRNA expression profile in the CD4 + T cells of SPMS patients; 42 dysregulated miRNAs were identified in the CD4 + T cells of SPMS patients when compared with healthy controls. Five of the miRNAs (miR-21-5p, -26b-5p, -29b-3p, -142-3p, and -155-5p) showed downregulated expression and possessed the potential to be used as a diagnostic SPMS biomarkers (Sanders et al., 2016). miR-1-3p expression is upregulated in Th17 cells in MS-relapse patients and is positively associated with the severity of MS. In addition, overexpression of miR-1-3p in naïve CD4 + T cells could promote Th17 cell differentiation by increasing the levels of inflammatory mediators; thus, knockout of miR-1-3p may be a potential therapeutic strategy for MS (Li et al., 2020). miR-590 is another highly expressed miRNA in Th17 cells of MS patients and could also promote Th17 cell differentiation through regulating IL-17A and RAR-related orphan receptor C expression. miR-590 may serve as a diagnostic biomarker and is a crucial target for potential therapeutic intervention (Liu Q. et al., 2017).

The recurrence rate of pregnant MS patients is 66% less than that of other MS patients (Li et al., 2021). In pregnant MS patients, differentially expressed miRNAs were performed with quantitative real-time PCR on PBMC. Flow cytometry analyzed on PBMC stained with antibodies directed against surface markers of antigen presenting cells (APCs), CD4+ and CD8 + T cells, NK-cells, NKT cells and subsets of these cell types, including programmed cell death 1 ligand 1 (PDL1) and PDL2 expressing subsets. The results showed that the expressions of miR-1, miR-20a, miR-28, miR-95, miR-146a, miR-335, and miR-625 in the PBMC of pregnant MS patients were downregulated, while the levels of PDL1, PDL2 and IL-10 were increased compared with untreated MS patients. The PDL1, PDL2, and IL-10 were the illustrated targets of these miRNAs and there was a negative correlation between them (Søndergaard et al., 2020).

However, miRNAs as diagnostic biomarkers of MS are still a long way from their application in clinical practice. First, miRNAs derived from peripheral T lymphocytes with differential expression rarely overlap; each study has drawn different conclusions, making it impossible to determine the reference value of each miRNA. In addition, there is a lack of international agreement on normalization methods and unified references owing to the different detection methods used in each study. Even if most studies used RT-PCR to detect miRNA expression levels, the results differ owing to different amplification times in RT-PCR detection, and normalization methods may influence the results. Moreover, miRNA from different sources and measured in different moments may also differ (the expression of miRNA in serum could differ from the expression of miRNA in different cell subtypes). Finally, there is a lack of well-designed multicenter studies; for instance, Honardoost et al. (2014) suggested that miR-326 has 100% specificity and sensitivity in MS diagnosis, but this still needs to be validated in a multicenter-controlled study with a larger number of MS patients. Regardless, despite the many shortcomings, the development of miRNAs as biomarkers is critical for future drug development and a better understanding of disease pathogenesis. In addition, miRNAs differentially expressed in peripheral T lymphocytes are non-invasive or minimally invasive; as such, they remain attractive targets for the early application of miRNAs in T lymphocytes as diagnostic biomarkers for MS in clinical practice.

## MicroRNAs in T Lymphocytes as Multiple Sclerosis Therapeutic Targets

Studies on the intervention of the MS pathological mechanism by regulating miRNA expression in peripheral T lymphocytes are currently mainly focused on EAE models, in which regulating miRNA expression has a therapeutic effect. For instance, miR-301a expression is upregulated in the T cells of EAE models which promotes Th17 cell differentiation; knockout miR-301a can inhibit Th17 cell development by targeting the IL-6/23-STAT3 pathway, and thus miR-301a expression may be a therapeutic target for controlling autoimmune demyelination (Mycko et al., 2012). Suppression of miR-155 can decrease Th1 and Th17 responses in the CNS, and reduce the clinical severity of EAE, even before the appearance of clinical symptoms (Murugaiyan et al., 2011). In an *in vitro* model of MS, inhibited miR-155 expression reduced the expression of CD4 + T cell effector cytokines IFN-γ and IL-17, contributing to the view that miR-155 might be a valuable target in MS therapy (Jevtić et al., 2015). miR-467b expression was decreased in CD4 + T cells of EAE; silencing miR-467b could inhibit the differentiation and function of Th17 cells by targeting eIF4E, which would alleviate EAE (Wu et al., 2021). Lentiviral vectors for miR-20b overexpression *in vivo* also led to decreased Th17 cells and reduced severity of EAE (Zhu et al., 2014).

In addition to regulating the expression of Th17 cell-related miRNAs to treat MS, modulation of the Th2 to Th1 shift is also a major research direction. The expression of miR-27b, miR-128, and miR-340 is upregulated in CD4 + T cells from MS patients, which induces the shift from Th2 to Th1 cytokines. Oligonucleotide-miRNA inhibitors of these miRNAs have been shown to induce the restoration of Th2 responses *in vitro*. *In vivo* experiments also suggest that silencing miR-27b, miR-128, and miR-340 could be used to treat MS patients (Guerau-De-Arellano et al., 2011).

However, current studies confirming the potential therapeutic effects of regulating T-cell-associated miRNAs on MS are limited to *in vitro* or in EAE models; there are many challenges in applying this strategy in clinical settings. (i) Although miRNAs are designed to target and regulate specific T-cell-related genes, miRNAs may also target genes unrelated to therapeutic effects and may produce unexpected changes in gene expression levels. Therefore, precise regulation is key to the clinical application of miRNA agomir/antagomir drugs. (ii) As most animal studies of miRNA therapeutic strategies focus on target tissues of interest, it is possible to overlook the off-target effects of miRNA agomir/antagomir in other tissues. How to avoid off-target effect is an important research direction of miRNA therapeutic strategies. (iii) Standardized dosage and schedule, efficacy verification, and prevention of side effects will also be difficulties that need to be overcome in the study of miRNAs as a treatment strategy.

## Conclusion

MicroRNAs play an important role in fine-tuning the adaptive immune response to inflammatory factors and MS. They can also directly regulate the involvement of T lymphocytes in the pathological mechanism of MS. Although emerging studies have determined the differential expression of miRNAs in a variety of T lymphocytes from MS patients, there is still a lot of work in progress regarding the mechanism of miRNA networks and their targets. Better understanding of how miRNA expression affects T lymphocyte responses would provide clues to the potential relevance of a given miRNA during the disease or allow identification of biomarkers for the discrimination of MS subtypes. However, being able to transform from association to causation is a difficult and challenging task. Only by identifying and verifying the mRNAs specifically targeted by each relevant miRNA can it be determined which miRNAs should be considered as effective options for MS treatment.

## Author Contributions

LW performed the literature searches and wrote the manuscript. YL critically revised the manuscript. Both authors listed have made a substantial, direct, and intellectual contribution to the work, and approved it for publication.

## Conflict of Interest

The authors declare that the research was conducted in the absence of any commercial or financial relationships that could be construed as a potential conflict of interest.

## Publisher’s Note

All claims expressed in this article are solely those of the authors and do not necessarily represent those of their affiliated organizations, or those of the publisher, the editors and the reviewers. Any product that may be evaluated in this article, or claim that may be made by its manufacturer, is not guaranteed or endorsed by the publisher.
